# Carnosine suppresses neuronal cell death and inflammation induced by 6-hydroxydopamine in an *in vitro* model of Parkinson's disease

**DOI:** 10.1371/journal.pone.0240448

**Published:** 2020-10-14

**Authors:** Maho Kubota, Nahoko Kobayashi, Toshifumi Sugizaki, Mikako Shimoda, Masahiro Kawahara, Ken-ichiro Tanaka

**Affiliations:** Laboratory of Bio-Analytical Chemistry, Research Institute of Pharmaceutical Sciences, Faculty of Pharmacy, Musashino University, Tokyo, Japan; Toho University Graduate School of Medicine, JAPAN

## Abstract

Parkinson's disease is a progressive neurodegenerative disease for which prevention and effective treatments are lacking. The pathogenesis of Parkinson's disease is not clearly understood. It is thought to be caused by oxidative stress-dependent loss of dopamine neurons in the substantia nigra and the promotion of inflammatory responses by microglia at the lesion site. In addition, cell loss occurs in the hypothalamus of Parkinson's disease patients. Carnosine is an endogenous dipeptide that can exert many beneficial effects, including an antioxidant action, metal ion chelation, proton buffering capacity, and inhibition of protein carbonylation and glycolysis. Previously, we found that carnosine inhibits trace metal-induced death of immortalized hypothalamic neuronal GT1-7 cells. In this study, we analyzed the efficacy of carnosine on 6-hydroxydopamine (6-OHDA)-dependent GT1-7 cell death and inflammatory responses. We found that carnosine significantly prevented 6-OHDA-dependent GT1-7 cell death in a dose-dependent manner. Moreover, carnosine significantly suppressed the expression of 6-OHDA-induced integrated stress response (ISR)-related factors and pro-inflammatory cytokines. Carnosine also significantly inhibited 6-OHDA-dependent reactive oxygen species (ROS) production and c-Jun amino-terminal kinase (JNK) pathway activation in GT1-7 cells. These results indicate that carnosine inhibits hypothalamic neuronal cell death and inflammatory responses by inhibiting the ROS-JNK pathway. We therefore suggest that carnosine may be effective in preventing the onset or the exacerbation of Parkinson's disease.

## 1. Introduction

Parkinson's disease is a progressive neurodegenerative disease that presents with motor deficits such as resting tremor, muscle rigidity, akinesia, and impaired postural reflexes. As Parkinson's disease progresses, people may have difficulty walking on their own and become wheelchair-bound or bedridden. Age is the main risk factor for Parkinson's disease and it develops in people over the age of 60 at a rate of about 1 in 100 [1, 2]. The number of dopamine neurons in the brain progressively decreases in Parkinson's patients; therefore, the dopamine precursor, L-dopa, anticholinergics, and amantadine hydrochloride are used for treatment [3]. However, no methods for prevention or effective long-term treatment of Parkinson's disease have been established.

Although the pathogenesis of Parkinson's disease is not clearly understood, it is thought to be caused by loss of dopamine neurons in the substantia nigra and the promotion of inflammatory responses by microglia at the lesion site [4, 5]. Dopaminergic neuronal shedding and microglial activation have also been observed in animal models of Parkinson's disease established using 6-hydroxydopamine (6-OHDA) or 1-methyl-4-phenyl-1,2,3,6-tetrahydropyridine (MPTP) [6]. Dysfunction of the hypothalamo-pituitary-adrenal (HPA) axis is also considered to be involved in Parkinson's disease pathogenesis [7]. The hypothalamus regulates endocrine and autonomic functions. Lower plasma levels of adrenocorticotropic hormone (ACTH) and cortisol have been recorded in Parkinson's disease patients compared to healthy controls [8]. Moreover, cell loss has also been observed in the hypothalamus of Parkinson's disease patients [9]; Thannickal et al. reported 50% hypothalamic cell loss in 10 patients with Parkinson's disease, with cell loss increasing with severity [10]. Therefore, we speculate that inhibiting neuronal cell death and inflammatory responses in the hypothalamus may be important to prevent the development or exacerbation of Parkinson's disease.

Carnosine is an endogenous dipeptide composed of β-alanine and L-histidine that is synthesized by carnosine synthase. Commonly consumed foods such as beef, pork, and fish contain carnosine. Carnosine has many beneficial effects, including antioxidant action, metal ion chelation, proton buffering capacity, and inhibition of protein carbonylation and glycolysis [11–13]. Many studies have shown that carnosine has neuroprotective effects against various neurological diseases, such as the neurodegenerative diseases, prion disease and Alzheimer's disease, and autism spectrum disorders and Gulf War Syndrome [13, 14]. In addition, we have reported that carnosine inhibits cell death and integrated stress responses (ISR) in hypothalamic neurons caused by excessive amounts of trace metals [15–17], indicating that it may be effective in the treatment of neurodegenerative diseases.

Several reports have examined the efficacy of carnosine in Parkinson's disease, especially and the efficacy of carnosine in animal models of Parkinson's disease has been shown. Daily intranasal administration of carnosine improved Parkinson's-like symptoms and reduced the accumulation of alpha-synuclein in Thy1-αSyn transgenic mice, a mouse model of Parkinson's disease [18]. In addition, carnosine pretreatment markedly reduced MPTP-induced oxidative stress and proinflammatory cytokine production in a mouse MPTP-induced model of Parkinson's disease [19]. In a clinical pilot study, carnosine administered as a food additive as part of a basic protocol for the treatment of Parkinson's disease significantly improved neurological symptoms and increased erythrocyte Cu/Zn-SOD levels and decreased plasma protein carbonyl and lipid hydroperoxide levels [20]. These results indicate that carnosine may be useful in the treatment and prevention of Parkinson's disease. However, the effects of carnosine on the inflammatory response and death of hypothalamic neurons in a Parkinson's disease model have not been reported.

In the present study, we analyzed the inhibition of 6-OHDA-dependent neuronal cell death and inflammatory responses by carnosine using GT1-7 cells, which are immortalized hypothalamic neurons. We focused on oxidative stress as the mechanism by which carnosine inhibits 6-OHDA-dependent neuronal death and inflammatory responses.

## 2. Methods

### 2.1. Chemicals and reagents

Carnosine was kindly provided by Hamri Chemicals, Ltd. (Osaka, Japan). 6-OHDA was purchased from R&D Systems, Inc. (Minneapolis, MN). CellTiter-Glo^®^ 2.0 and ROS-Glo^TM^ were purchased from Promega Corporation (Madison, WI). Dulbecco’s Modified Eagle’s Medium (DMEM)/Ham’s Nutrient Mixture F-12 (D-MEM/Ham’s-F12) was purchased from WAKO Pure Chemicals (Tokyo, Japan). RNeasy kits were purchased from Qiagen (Hilden, Germany). PrimeScript^®^ 1st strand cDNA Synthesis Kits were purchased from Takara Bio (Ohtsu, Japan). THUNDERBIRD^®^ SYBR qPCR Mix was purchased from Toyobo (Osaka, Japan). APOPCYTO Caspase Colorimetric Assay Kits were from Medical & Biological Laboratories Co., Ltd. (Nagoya, Japan).

### 2.2. Cell culture

Mouse GT1-7 cells (provided by Dr. R. Weiner, University of California, San Francisco) were grown in DMEM/Ham’s-F12 supplemented with 10% fetal bovine serum. After trypsin digestion, cells were resuspended in serum-free medium, distributed into culture dishes and cultured in a humidified incubator (7% CO_2_) at 37°C [21].

### 2.3. Measurement of viable cell number and detection of apoptosis

Viable cell number was measured as previously described [22, 23]. Briefly, dissociated GT1-7 cells were distributed into 96-well culture plates at a concentration of 2.0 × 10^4^ cells per well in 200 μL culture medium. After incubation for 24 h, cells were treated with carnosine (0–8 mM) and 6-OHDA. After exposure for 24 h, viable cell number was quantified using CellTiter-Glo^®^ 2.0.

To detect apoptotic cell death, cells were prepared as described above. After incubation for 24 h, cells were treated with carnosine (0–8 mM) and 6-OHDA for 8 h. Cells were then harvested and apoptotic cell death was measured using the APOPCYTO Caspase Colorimetric Assay Kit.

### 2.4. Measurement of reactive oxygen species (ROS) levels

GT1-7 cells were pre-cultured in black 96-well microplates (2.0 × 10^4^ cells/well) for 24 h. Cells were then treated with carnosine (0–8 mM) and 6-OHDA for 1 h and the levels of ROS were then quantified using ROS-Glo^TM^ (Promega Corporation, Madison, WI).

### 2.5. Real-time RT-PCR

Total RNA was extracted from GT1-7 cells grown in 6-well culture plates (5.0 × 10^5^ cells per well in 1.5 mL culture medium) using an RNeasy kit as previously described [24, 25]. RNA samples were reverse-transcribed using the PrimeScript^®^ 1st strand cDNA Synthesis Kit. Real-time RT-PCR was performed using the cDNAs as templates with THUNDERBIRD^®^ SYBR qPCR Mix on the Bio-Rad CFX96™ Real-time system with CFX Manager™ software (Hercules, CA). Specificity was confirmed by electrophoretic analysis of reaction products and by the inclusion of template- or reverse transcriptase-free controls. To normalize the amount of total RNA present in each reaction, glyceraldehyde-3-phosphate dehydrogenase (*Gapdh*) cDNA was used as an internal standard. Primers were designed using Primer-BLAST. Primers sequences will be provided upon request.

### 2.6. Western blotting

Levels of phospho-SAPK/JNK (p46 and p54), and actin were assessed by western blotting. GT1-7 cells grown in 6-well culture plates (5.0 × 10^5^ cells per well) were lysed with radioimmunoprecipitation [26] buffer containing both protease and phosphatase inhibitors (Code: 87786 and 78420, Thermo Fisher Scientific Inc). Protein concentration was measured using Bradford Reagent (Bio-Rad, Hercules, CA, USA). Samples were applied to NuPAGE^®^ Novex 4–12% Bis-Tris Protein Gels (Thermo Fisher Scientific Inc.), electrophoresed at a constant voltage of 180 V, and proteins transferred to polyvinylidene difluoride (PVDF) membranes using the iBlot^®^ 7-Minute Blotting System (Thermo Fisher Scientific Inc.). Membranes were blocked with 5% non-fat dried milk at room temperature for 1 h, and incubated overnight with a rabbit anti-phospho-SAPK/JNK antibody (1:1,000 dilution) or a mouse anti-actin antibody (1:1,000 dilution) in 5% bovine serum albumin (BSA), 1× Tris-buffered saline (TBS) and 0.1% Tween-20, followed by incubation with a goat anti-rabbit IgG, HRP-linked antibody (1:2,000 dilution) or a horse anti-mouse IgG, HRP-linked antibody (1:4,000 dilution) in 1× TBS and 0.1% Tween-20 for 1 h. Protein bands were visualized using SuperSignal™ West Dura Extended Duration Substrate (Thermo Fisher Scientific Inc.). Band intensities were quantitated using ImageJ software (version 1.39u). The band intensity of each protein was determined and normalized with respect to actin intensity.

### 2.7. Statistical analysis

All data are expressed as the mean ± S.E. Significant differences between groups were examined using one-way of analysis of variance (ANOVA) followed by Tukey’s multiple comparison. SPSS24 software was used for all statistical analyses. P< 0.05 was considered to indicate statistical significance.

## 3. Results

### 3.1. Induction of neuronal cell death by 6-OHDA treatment

**Fig 1:**
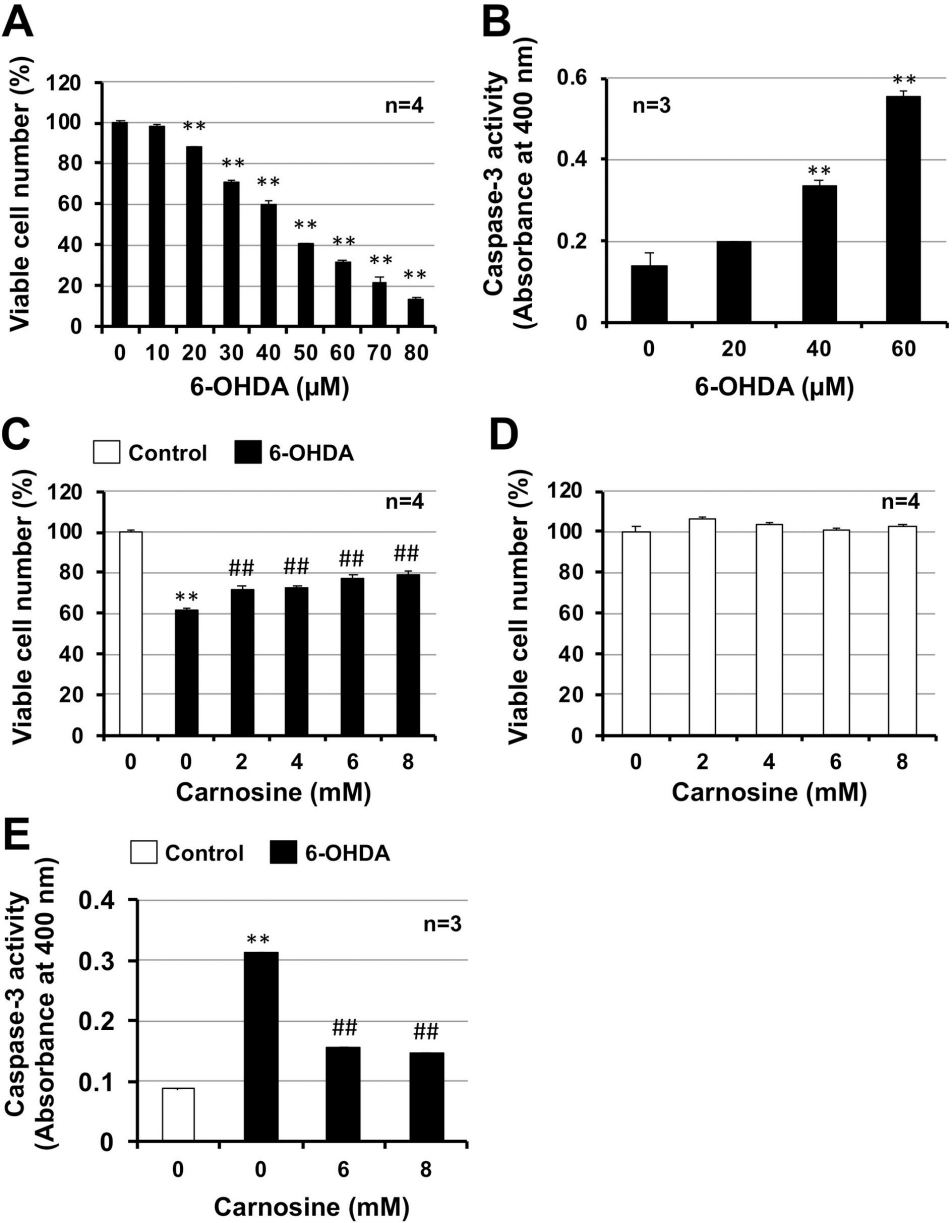
GT1-7 cells were incubated with the indicated concentrations of 6-OHDA for 24 h (A) or 8 h (B). GT1-7 cells were pre-treated with the indicated concentrations (mM) of carnosine and were then incubated in the absence (Control) or presence of 6-OHDA (40 μM) for 24 h (C) or 8 h (D). GT1-7 cells were incubated with the indicated concentrations (mM) of carnosine for 24 h (D). Viable cell number was determined using CellTiter-Glo^®^ 2.0 (A, C, D). Caspase-3 activity was detected using the APOPCYTO Caspase Colorimetric Assay Kit (B, E). Values represent the mean ± S.E. ** P<0.01 (vs Control).

6-OHDA causes Parkinson's disease-like symptoms in animals and induces neuronal cell death *in vitro* [6, 27]. In this study, we first analyzed 6-OHDA-induced neuronal cell death in GT1-7 cells. We measured intracellular ATP levels using CellTiter-Glo^®^ 2.0 to determine cell viability. As shown in Fig 1A, 6-OHDA (0–80 μM) dose-dependently decreased intracellular ATP levels of GT1-7 cells. Cell viability after treatment with 40, 50, or 60 μM 6-OHDA was 59.8 ± 1.6, 40.5 ± 0.2, and 31.1 ± 1.2% (mean ± S.E., n = 4) of control, respectively. We then measured caspase-3 activity in GT1-7 cells to monitor apoptotic cell death. As shown in Fig 1B, 6-OHDA (0–60 μM) treatment increased caspase-3 activity in a dose-dependent manner. These results indicate that 6-OHDA induces hypothalamic neuronal cell death.

### 3.2. Effect of carnosine on 6-OHDA-induced neuronal cell death

We previously reported that carnosine suppressed Zn^2+^-induced neuronal cell death. Carnosine may therefore be effective for treating neurodegenerative diseases [12, 15, 16]. We examined the effect of carnosine on 6-OHDA-induced neuronal cell death in GT1-7 cells. As shown in Fig 1C, treatment of GT1-7 cells with 6-OHDA (40 μM) decreased intracellular ATP levels. The viability of cells exposed to 6-OHDA (40 μM) was 61.4 ± 1.3% (mean ± S.E., n = 4). In contrast, carnosine treatment (0–8 mM) significantly restored 6-OHDA-decreased intracellular ATP levels in GT1-7 cells in a dose dependent manner. The viability of cells exposed to 6-OHDA plus carnosine (6, 8 mM) was 77.5 ± 1.8 and 79.2 ± 1.4% (mean ± S.E., n = 4), respectively. Moreover, 6-OHDA-induced apoptotic cell death measured by caspase-3 activity was clearly suppressed by carnosine treatment (Fig 1E). Under these conditions, carnosine treatment alone (0–8 mM) did not affect the number of viable GT1-7 cells (Fig 1D). These results indicate that carnosine suppressed 6-OHDA-induced hypothalamic neuronal cell death.

### 3.3. Effect of carnosine on 6-OHDA-induced integrated stress responses

**Fig 2:**
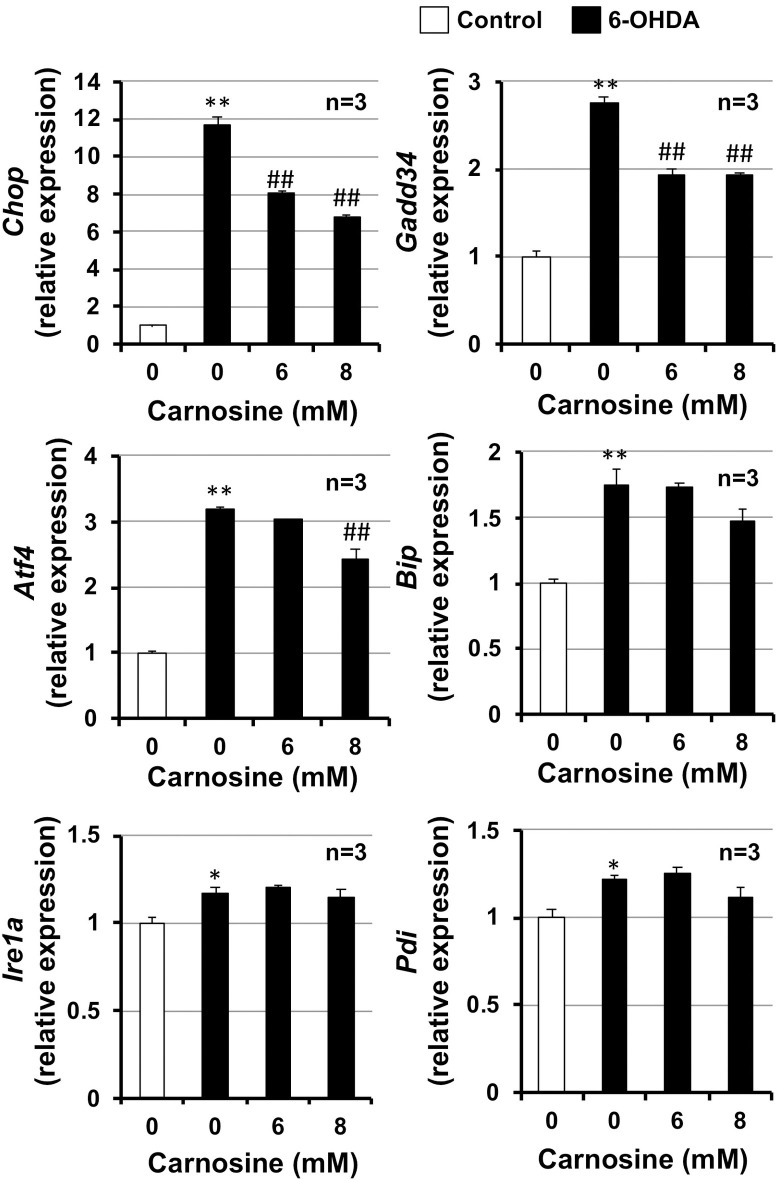
GT1-7 cells were incubated with the indicated concentrations (mM) of carnosine and then in the absence (Control) or presence of 6-OHDA (40 μM) for 6 h. Total RNA was extracted from GT1-7 cells and subjected to real-time RT-PCR using primer sets specific for *Chop*, *Gadd34*, *Atf4*, *Bip*, *Ire1a*, and *Pdi*. Values were normalized to *Gapdh* and are expressed relative to the control. Values represent the mean ± S.E.M. *P<0.05, ** or ^##^ P<0.01 (* vs Control, ^#^ vs 6-OHDA alone).

Carnosine attenuates stress-induced neuronal cell death by suppressing the integrated stress response [12, 15, 16]. The integrated stress response is a mechanism of 6-OHDA-dependent apoptosis [28, 29]; therefore, we next examined the effect of carnosine on the 6-OHDA-dependent integrated stress response. As shown in Fig 2, integrated stress-related genes, such as CCAAT-enhancer-binding protein homologous protein (*Chop*), growth-arrest and DNA-damage-inducible gene 34 (*Gadd34*), activating transcription factor 4 (*Atf4*), heat shock protein 5 (*Hspa5*) (also known as *Bip*), ER to nucleus signaling 1 (*Ern1*) (also known as *Ire1a*), and protein disulfide isomerase [30] were induced by 6-OHDA treatment. In contrast, carnosine (6, 8 mM) significantly suppressed the induction of *Chop*, *Gadd34*, and *Atf4* mRNAs by 6-OHDA treatment, but did not suppress the induction of *Bip*, *Ire1a*, or *Pdi* mRNAs. These results indicate that carnosine suppresses 6-OHDA induced hypothalamic neuronal cell death by suppressing integrated stress responses.

### 3.4. Effect of carnosine on 6-OHDA-induced inflammatory responses

**Fig 3:**
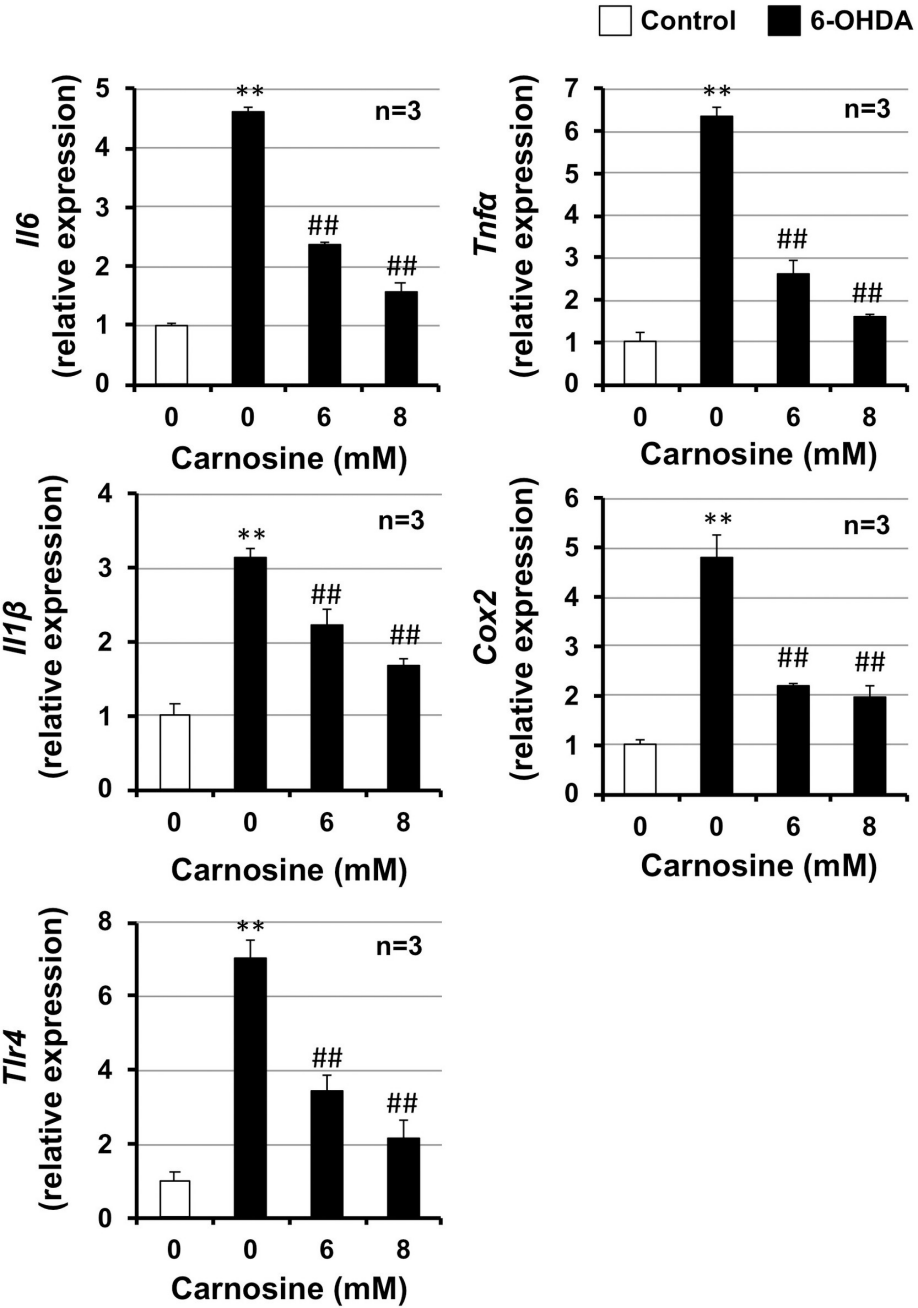
GT1-7 cells were incubated with the indicated concentrations (mM) of carnosine and then in the absence (Control) or presence of 6-OHDA (40 μM) for 6 h. Total RNA was extracted from GT1-7 cells and subjected to real-time RT-PCR using primer sets specific for *Il6*, *Tnfa*, *Il1b*, *Cox2*, and *Tlr4*. Values were normalized to *Gapdh* and are expressed relative to the control. Values represent the mean ± S.E.M. *P<0.05, ** or ^##^ P<0.01 (* vs Control, ^#^ vs 6-OHDA alone).

Inflammatory responses possibly cause the onset of Parkinson's disease and exacerbate its severity [5]. Carnosine exerts anti-inflammatory effects in a variety of inflammatory models [13, 31, 32]. We therefore examined whether carnosine suppresses 6-OHDA-induced inflammatory responses under our experimental conditions. As shown in Fig 3, pro-inflammatory cytokines, such as interleukin 6 (*Il6*), tumor necrosis factor alpha (*Tnfa*), *Il1b*, cyclooxygenase 2 (*Cox2*), and toll-like receptor 4 (*Tlr4*) were induced by 6-OHDA treatment, while carnosine treatment significantly suppressed this up-regulation. These results indicate that carnosine suppressed 6-OHDA-induced inflammatory responses in hypothalamic neuronal cells.

### 3.5. Mechanisms by which carnosine inhibits cell death and inflammation

**Fig 4:**
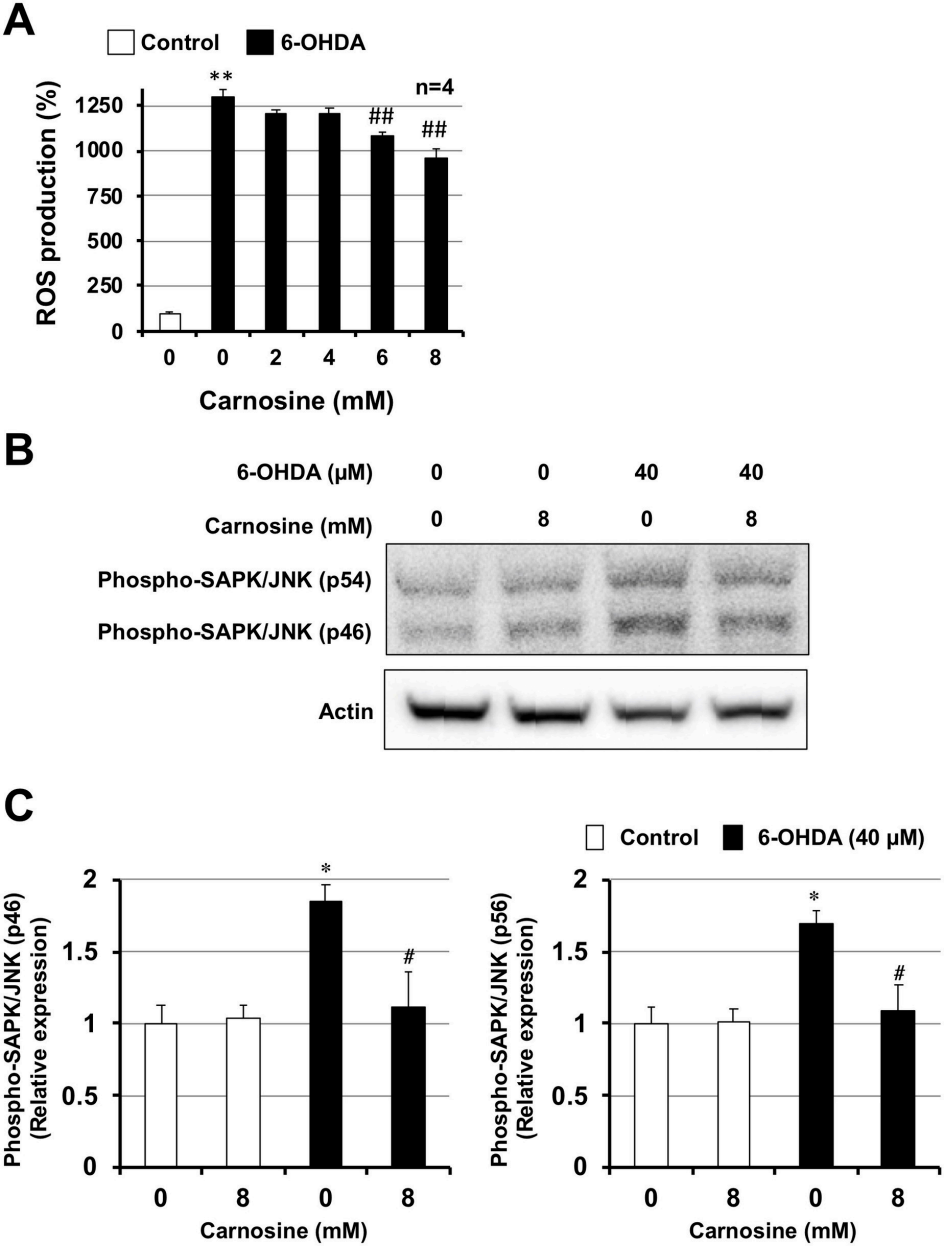
GT1-7 cells were incubated with the indicated concentrations (mM) of carnosine and then in the absence (Control) or presence of 6-OHDA (40 μM) for 2 h (A–C). ROS levels were quantified using ROS-Glo^TM^ (A). Whole-cell extracts were analyzed by immunoblotting using an antibody against phospho-SAPK/JNK, or actin (B). Band intensity of phospho-SAPK/JNK was determined using Image J software (C). Values represent the mean ± S.E.M. * or ^#^P<0.05, ** or ^##^ P<0.01 (* vs Control, ^#^ vs 6-OHDA alone).

6-OHDA induces neuronal death through ROS production [33, 34] and ROS production may cause the onset of Parkinson's disease and exacerbate its severity [35]. Carnosine has antioxidant properties [11], therefore, we examined whether 6-OHDA induces ROS production under our experimental conditions using the ROS-Glo^TM^ assay kit. As shown in Fig 4A, 6-OHDA (40 μM) clearly induced ROS production in GT1-7 cells. However, the ROS production induced by 6-OHDA was significantly suppressed by carnosine treatment, indicating that carnosine inhibits 6-OHDA-induced hypothalamic neuronal cell death and inflammatory responses by inhibiting ROS production.

Stress-activated protein kinases/c-Jun amino-terminal kinases (SAPKs/JNKs) are members of the mitogen-activated protein kinase (MAPK) family, and the SAPK/JNK signaling pathway plays an important role in apoptosis, necroptosis, and inflammation [36]. Furthermore, the SAPK/JNK signaling pathway is involved in the development of Parkinson’s disease [37]. We therefore examined the effect of carnosine on 6-OHDA-induced activation of SAPK/JNK signaling using western blotting. Treatment of GT1-7 cells with 6-OHDA (40 μM) significantly increased levels of phospho-JNK (p46) and phospho-JNK (p54) at 2 h after treatment (Fig 4B and 4C). In contrast, carnosine treatment (8 mM) significantly suppressed the levels of these phospho-proteins (Fig 4B and 4C). Finally, we examined the effect of SP600125 (an inhibitor of the SAPK/JNK signaling pathway) on 6-OHDA-induced hypothalamic neuronal cell death and inflammatory responses. As shown in S1A Fig, SP600125 significantly restored 6-OHDA-decreased intracellular ATP levels in GT1-7 cells in a dose dependent manner. Under these conditions, SP600125 treatment alone did not affect the number of viable GT1-7 cells (S1B Fig). Moreover, SP600125 treatment significantly suppressed the induction of *Chop* and *Il6* mRNAs by 6-OHDA treatment (S1C Fig). These results indicate that carnosine inhibits hypothalamic neuronal cell death and inflammatory responses by inhibiting the ROS-JNK pathway.

## 4. Discussion

In the present study, we demonstrated that 6-OHDA-dependent hypothalamic neuronal cell injury and promotion of inflammatory responses are attenuated by carnosine treatment. We also found that carnosine attenuated the 6-OHDA-dependent integrated stress response. Specifically, carnosine markedly inhibited 6-OHDA-induced upregulation of the ISR-related genes, *Chop*, *Gadd34*, and *Atf4*. Cellular stress signals (such as ER stress and viral infection) activate protein kinase R-like ER kinase, RNA-activated protein kinase, heme-regulated inhibitor, and general control non-derepressible 2 that converge on phosphorylation of eukaryotic translation initiation factor 2α (eIF2α), the core of ISR. This initiates preferential translation of ISR-specific mRNAs, such as ATF 4, while global cap-dependent translation is attenuated [38]. ATF4 then activates transcription of CHOP and GADD34, which are involved in cell death induction in various cells [39]. We therefore suggest that carnosine inhibits 6-OHDA-dependent hypothalamic neuronal cell death by inhibiting 6-OHDA-dependent integrated stress response.ROS and the downstream SAPK/JNK signaling pathway are important factors in the development and exacerbation of Parkinson's disease. Alam et al. analyzed the ROS-related DNA damage product, 8-hydroxyguanine (8-OHG), in control and Parkinson's disease brains using gas chromatography/mass spectrometry, and found that levels of 8-OHG tended to be elevated in Parkinson's disease [40]. Another group also showed that cytoplasmic 8-OHG immunoreactivity in substantia nigra neurons in both multiple system atrophy-Parkinsonian type and dementia with Lewy bodies patients was increased compared with controls [41]. Moreover, oxidative stress (thiobarbituric acid reactive substances and advanced oxidation protein products) and inflammatory markers, such as myeloperoxidase, were significantly elevated in the blood of Parkinson’s disease patients. In contrast, the ferric reducing ability of plasma and vitamin C were significantly lower in Parkinson’s disease patients [42]. In contrast, Wang et al. showed that the levels of phosphorylated JNK in peripheral blood lymphocytes were significantly increased in Parkinson's disease (p<0.001) [43]. Furthermore, in MPTP-induced Parkinson's disease models, both ROS production and JNK activation were observed *in vivo* (in C57BL/6 mice) and *in vitro* (in PC12 cells) [44]. These reports suggest that the ROS-JNK pathway is important for the development of Parkinson's disease. Carnosine almost completely suppressed JNK activation by 6-OHDA, while carnosine partially suppressed ROS production. We speculate that JNK activation by 6-OHDA is largely dependent on ROS production under the present experimental conditions, but we speculate that other signals activated by ROS could not be suppressed by carnosine treatment. Therefore, we assume that inhibitors of other ROS-induced pathways, in combination with carnosine, may more strongly inhibit 6-OHDA-induced hypothalamic neuronal cell death. I would like to conduct further experiments in the future.

Numerous studies have tested the preventive and therapeutic effects of antioxidants in Parkinson’s disease animal models and in human clinical trials. Randomized, double-blind, placebo-controlled, parallel-group pilot trials showed that the reduced form of CoQ_10_ decreased Unified Parkinson's Disease Rating Scale (UPDRS) scores in Parkinson’s disease patients compared with a placebo group [45]. Recently, Monti et al. assessed the clinical effects of N-acetylcysteine (NAC), a precursor to the natural antioxidant, glutathione, in Parkinson's disease patients. NAC significantly increased dopamine transporter binding in the caudate and putamen and significantly improved Parkinson's disease symptoms [46]. Moreover, in 6-OHDA-induced Parkinson's disease models, manganese superoxide dismutase reduced striatal lesions and loss of neuronal cell bodies in the substantia nigra [47]. These reports indicate that antioxidant therapies, such as carnosine, may prevent the onset or exacerbation of Parkinson's disease.

In summary, we found that carnosine reduced 6-OHDA-induced neuronal cell death and inflammatory responses of immortalized mouse hypothalamic neuronal cells. Furthermore, we found that carnosine suppressed 6-OHDA-induced activation of the SAPK/JNK signaling pathway by inhibiting ROS production. Carnosine may therefore be effective in preventing the onset and/or exacerbation of Parkinson's disease.

## Supporting information

S1 Fig GT1-7 cells were pre-treated with the indicated concentrations (μM) of SP600125 and were then incubated in the absence (Control) or presence of 6-OHDA (40 μM) for 24 h (A) or 6 h (C). GT1-7 cells were incubated with the indicated concentrations (μM) of SP600125 for 24 h (B). Viable cell number was determined using CellTiter-Glo® 2.0 (A, C). Total RNA was extracted from GT1-7 cells and subjected to real-time RT-PCR using primer sets specific for Chop, and Il6. Values were normalized to Gapdh and are expressed relative to the control. Values represent the mean ± S.E. * or #P<0.05, ** or ## P<0.01 (* vs Control, # vs 6-OHDA alone).
(JPG)

S1 Raw images Full gel scans for Fig 4. (PDF)

## Abbreviations

ANOVA: analysis of variance
ACTH: adrenocorticotropic hormone
ATF: activating transcription factor
CHOP: CCAAT/enhancer binding protein homologous protein
COX: cyclooxygenase
Ern1: ER to nucleus signaling 1
eIF: eukaryotic translation initiation factor
ER: endoplasmic reticulum
GADD34: growth arrest and DNA-damage-inducible 34
GAPDH: glyceraldehyde-3-phosphate dehydrogenase
6-OHDA: 6-hydroxydopamine
HPA: hypothalamo-pituitary-adrenal
Hspa: heat shock protein family A member
IL: interleukin
IRE1: inositol-requiring enzyme-1
MAPK: mitogen-activated protein kinase
MPTP: 1-methyl-4-phenyl-1,2,3,6-tetrahydropyridine
NAC: N-acetyl cysteine
8-OHG: 8-hydroxyguanine
PERK: protein kinase R-like ER kinase
PVDF: polyvinylidene difluoride
ROS: reactive oxygen species
SAPK/JNK: stress-activated protein kinases/c-Jun amino-terminal kinases
TBS: tris-buffered saline
Tlr4: toll-like receptor 4
TNF: tumor necrosis factor
UPDRS: Unified Parkinson's Disease Rating Scale
