# Supplementary figures and images for: Carnosine suppresses neuronal cell death and inflammation induced by 6-hydroxydopamine in an in vitro model of Parkinson's disease
Source: PLoS One. 2020 Oct 14;15(10):e0240448. doi: 10.1371/journal.pone.0240448 (PMC7556511; doi:10.1371/journal.pone.0240448)

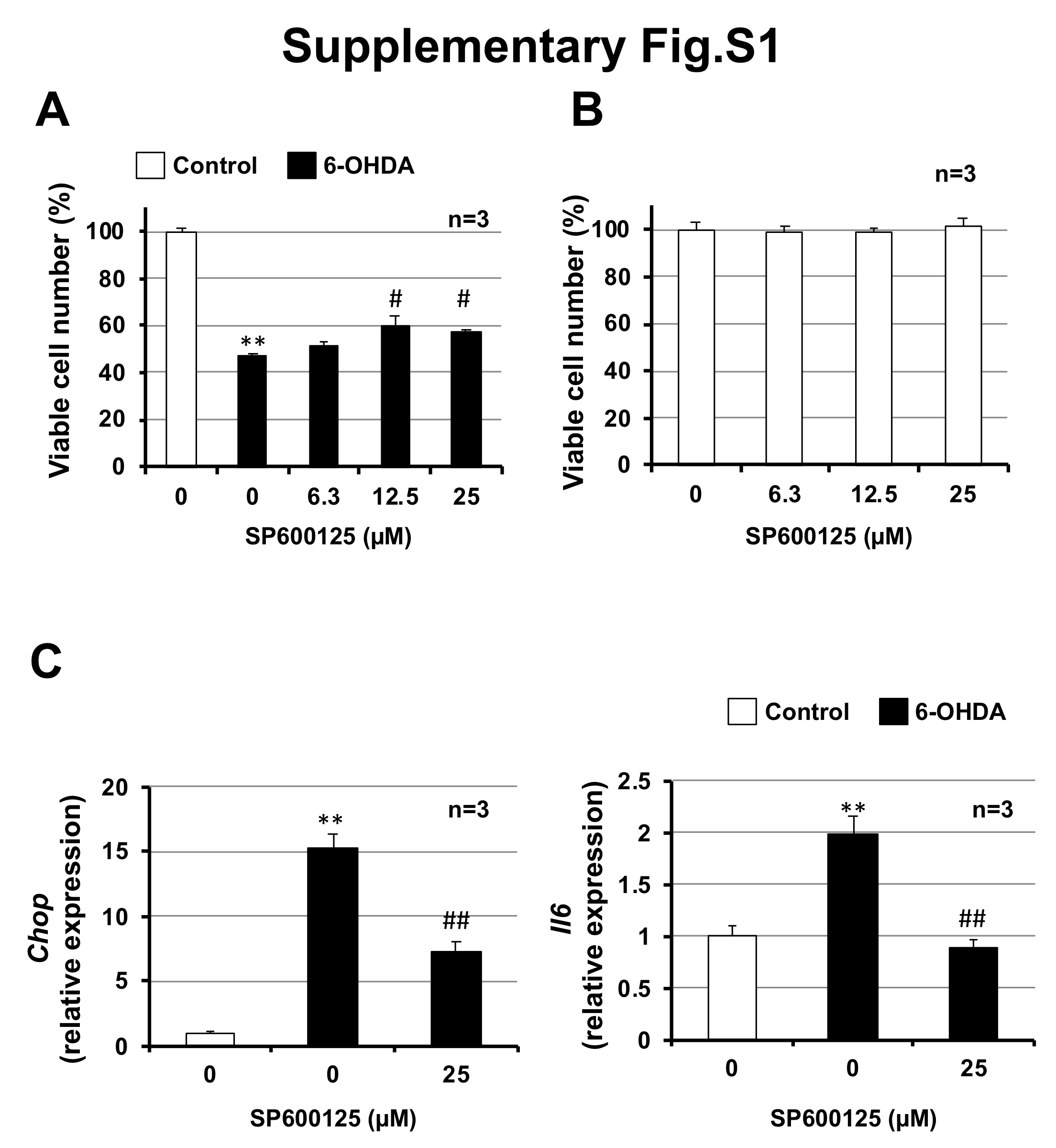

Supplement: S1 Fig — GT1-7 cells were pre-treated with the indicated concentrations (μM) of SP600125 and were then incubated in the absence (Control) or presence of 6-OHDA (40 μM) for 24 h (A) or 6 h (C). GT1-7 cells were incubated with the indicated concentrations (μM) of SP600125 for 24 h (B). Viable cell number was determined using CellTiter-Glo® 2.0 (A, C). Total RNA was extracted from GT1-7 cells and subjected to real-time RT-PCR using primer sets specific for Chop, and Il6. Values were normalized to Gapdh and are expressed relative to the control. Values represent the mean ± S.E. * or #P<0.05, ** or ## P<0.01 (* vs Control, # vs 6-OHDA alone). (JPG) [file pone.0240448.s001.jpg]

# Supplementary Fig.S2

**A**

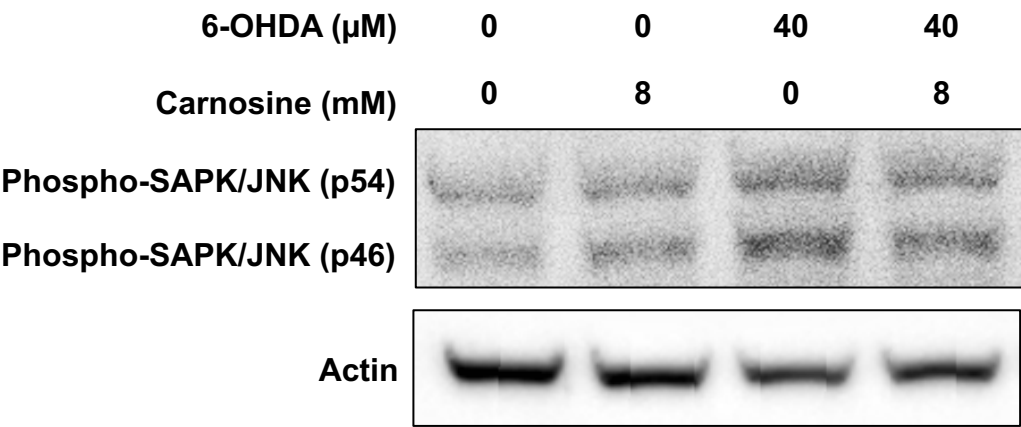

**B**

**Phospho-SAPK/JNK**

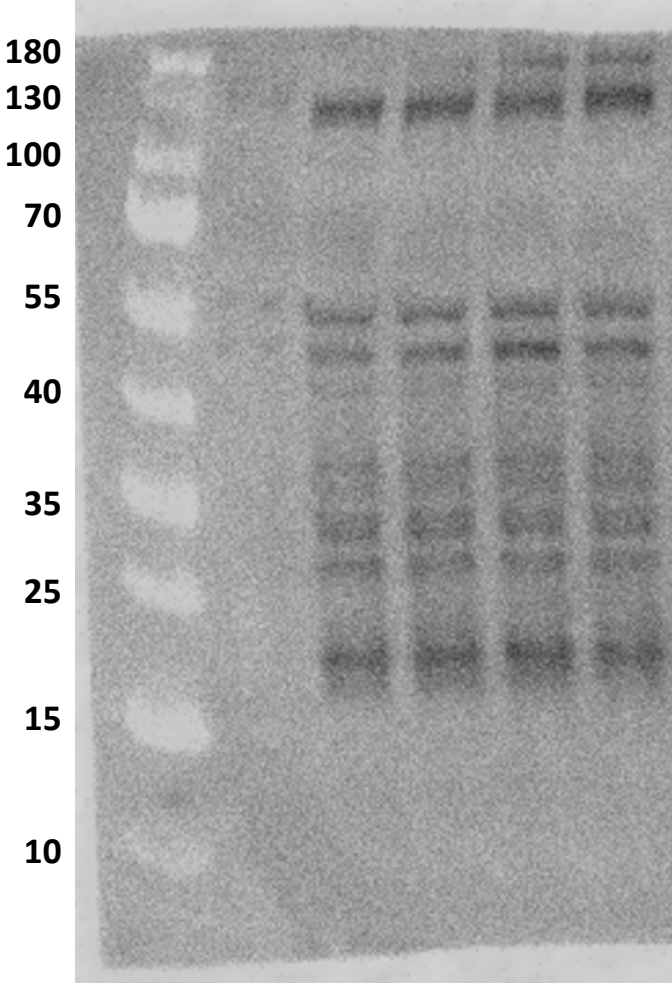

**Actin**

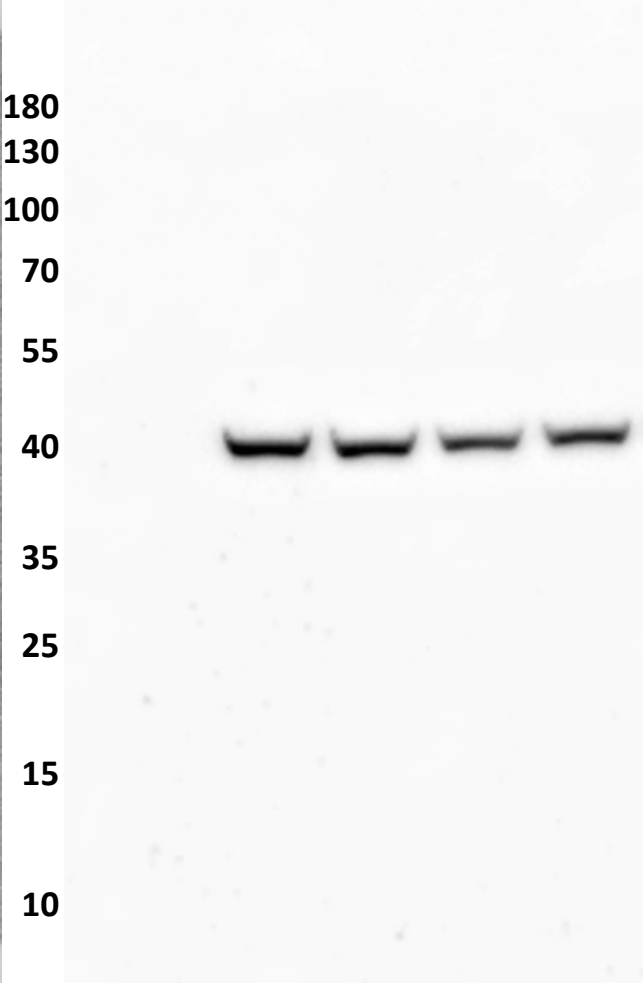

Supplement: S1 Raw images — (PDF) [file pone.0240448.s002.pdf]
